# Corneal proteome and differentially expressed corneal proteins in highly myopic chicks using a label-free SWATH-MS quantification approach

**DOI:** 10.1038/s41598-021-84904-4

**Published:** 2021-03-09

**Authors:** Byung Soo Kang, Thomas Chuen Lam, Jimmy Ka-wai Cheung, King Kit Li, Chea-su Kee

**Affiliations:** School of Optometry, The Hong Kong Polytechnic University, Hong Kong, SAR China

**Keywords:** Visual system, Proteomics, Biomarkers, Medical research

## Abstract

Myopia, or short-sightedness, is a highly prevalent refractive disorder in which the eye’s focal length is too short for its axial dimension in its relaxed state. High myopia is associated with increased risks of blinding ocular complications and abnormal eye shape. In addition to consistent findings on posterior segment anomalies in high myopia (e.g., scleral remodeling), more recent biometric and biomechanical data in myopic humans and animal models also indicate anterior segment anomalies (e.g., corneal biomechanical properties). Because the cornea is the anterior-most ocular tissue, providing essential refractive power and physiological stability, it is important to understand the biochemical signaling pathway during myopia development. This study first aimed to establish the entire chicken corneal proteome. Then, using the classical form deprivation paradigm to induce high myopia in chicks, state-of-the-art bioinformatics technologies were applied to identify eight differentially expressed proteins in the highly myopic cornea. These results provide strong foundation for future corneal research, especially those using chicken as an animal model for myopia development.

## Introduction

The cornea is a transparent, curved structure that occupies one-third of the eye shell and accounts for the majority of the refractive power of the eye. Approximately 80% of mass and volume of the cornea consists of a dense, interwoven collagen tissue, the stroma. Highly organized collagen fiber spacing with a homogenous diameter within the stroma ensures the clearness of light refraction and geometrical properties^1^. The primary focus of corneal research has been either managing disrupted collagen distributions caused by pathologies^2^ or refractive surgeries^3^ to restore visual clarity. However, there has been little interest in understanding corneal changes during refractive development. Despite the increasing myopia prevalence^4^ and potential ocular complications arising from high myopia development^5^, few studies have investigated the relationship between corneal geometries and the degree of refractive error in humans^6–10^.

While genomics has extended the understanding of etiology of diseases, it possesses a critical limitation in that mRNA levels may not fully reflect the level of final protein products^11,12^, mainly because of alternative splicing and post-translation modification (PTM)^13,14^. This problem led a natural movement from genomics to proteomics. However, comprehensive and universal proteome studies are limited due to the extremely dynamic properties of the proteome compared with the genome, which require highly sensitive and reproducible analytical methods. Compared to classic proteomics methods (e.g., gel electrophoresis) with restrictions of low throughput and specificity, mass spectrometry (MS) effectively facilitates the investigation of complex protein mixtures. Of the various types of MS available^15,16^, this study applied a hybrid quadrupole time-of-flight MS analytical proteomic technique^17,18^ integrated with the novel sequential windowed acquisition of all theoretical mass spectra (SWATH-MS)^18,19^. Using this approach, ionized peptides within the given range of the entire mass to charge ratio (m/z) are fragmented and recorded systematically in an unbiased fashion (data independent acquisition; DIA)^18,20^. Protein/peptide identification and quantification are performed in a targeted approach based on the prerequisite ion spectral libraries collected by information dependent acquisition (IDA), which contain intensities, m/z, and retention time of all precursors and their corresponding ion fragments^21^. Since proteins that are not listed in the ion spectral libraries cannot be analyzed and quantified, generating a comprehensive library that covers the extensive range of protein pools is crucial^22^. There have been successful investigations of the human corneal proteome^23–25^, but the chicken corneal proteome was not available because their corneas have not been used for proteomics-based research, unlike other ocular tissues such as the retina and vitreous^26,27^. In a pilot study, a total of 1214 corneal proteins were identified (unpublished) as an ion library from untreated chicken eyes. However, this ion spectral library had fundamental limitations: (1) the corneal proteins that appeared exclusively in myopic eyes were unquantifiable as they were not listed; and (2) low-abundant proteins might have been masked by plentiful proteins (e.g., collagens). To overcome these limitations, this study included corneas from highly myopic eyes as well as untreated control eyes for generating an in-depth ion spectral library using an offline peptide fractionation technique^28^. By integrating the generated library, differentially expressed corneal proteins in myopic eyes could be screened by applying SWATH-MS coupled to bioinformatics^29^.

Several animal models are used for myopia research^30–34^, but chicken is a particularly effective model for cornea studies^35^. Firstly, the chicken cornea is composed of five distinct layers similar to those in the human cornea, while other animals, such as rabbits and rodents lack the Bowman’s layer^36,37^. Secondly, although chickens have a slightly thinner cornea than humans, the relative thickness ratio is very similar^37^. In addition to similarity of anatomical features, the corneal morphology of chickens is responsive to various visual manipulations^38–40^ and lighting conditions^41,42^, which support the use of chicken corneas over those of other species.

## Results

**Figure 1:**
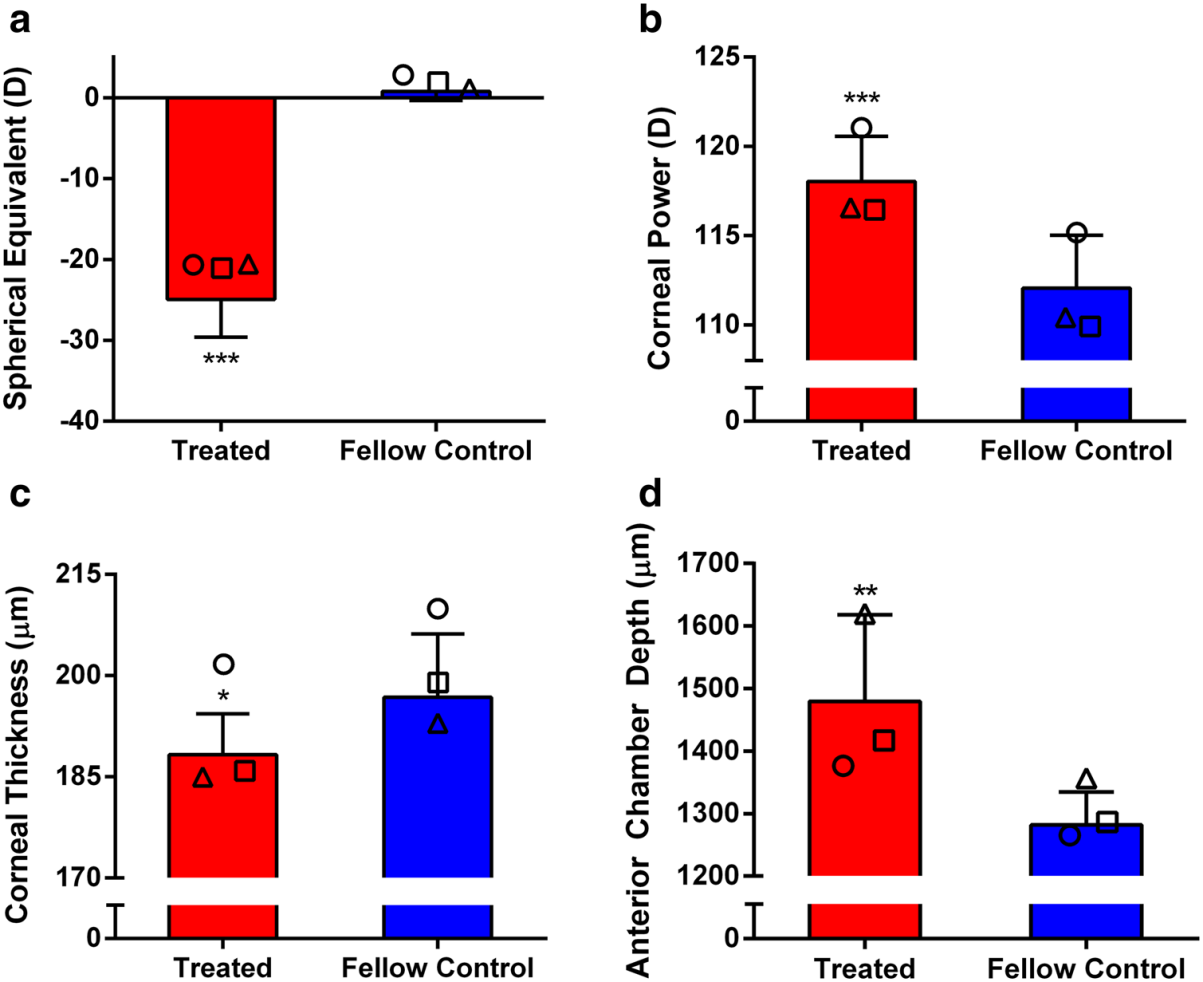
Ocular biometric parameters of eight chicks after a week of FD treatment. Treated eyes developed extremely high myopia (**a**) with a steeper cornea (**b**), a thinner cornea (**c**), and a deeper anterior chamber depth (**d**). Three of these chicks were used for proteomic analysis (Data from the same bird are represented with the same symbol). Paired *t*-test was performed on all parameters except central corneal thickness (Mann–Whitney *U*-test), *p < 0.05, **p < 0.01, ***p < 0.001. Bars represent mean ± SD.

**Figure 2:**
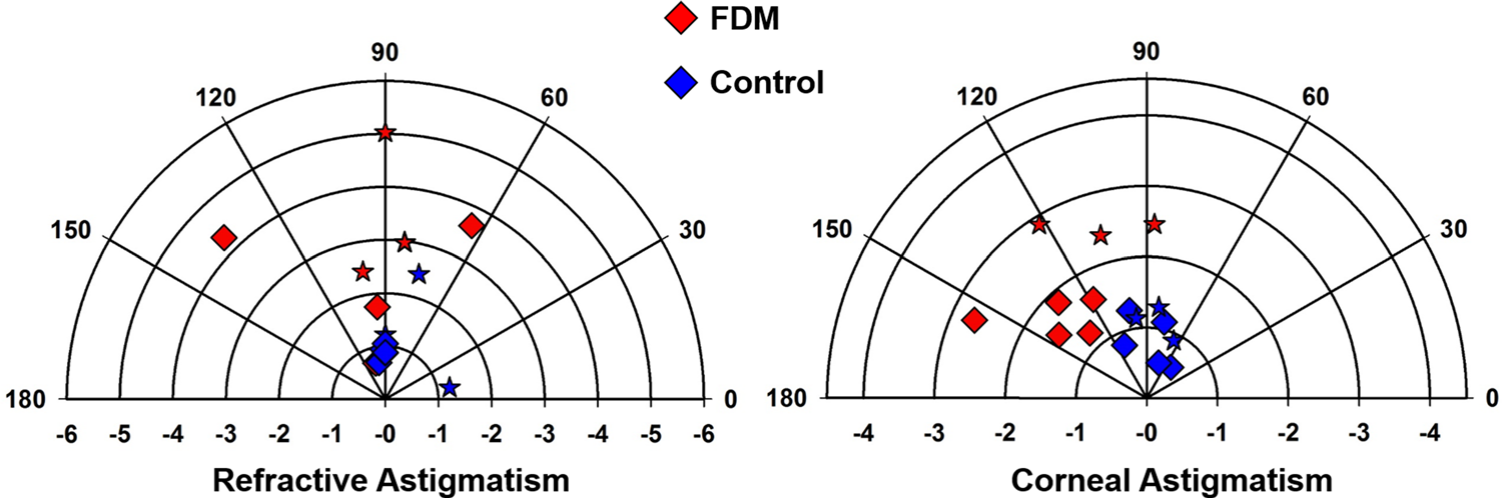
The distributions of refractive and corneal astigmatism in all eight FD-treated chicks. Each symbol in the polar plot represents the magnitude (radius) and axis (angle) of refractive (left) and corneal (right) astigmatism for one bird. Star symbols (★) indicate the eyes used for proteomic analysis.

As expected from the previous study^43^, form deprivation (FD) treatment for 7 days induced extremely high myopia (Fig. 1a) accompanied by significant refractive and corneal astigmatisms (Fig. 2) in the right eyes compared to fellow control eyes (mean ± SD: –24.91 ± 4.66 D vs. 0.79 ± 1.15 D; paired *t*-test, p < 0.001). Significant anterior ocular biometric changes were observed in the highly myopic eyes: deeper anterior chamber depth (mean ± SD: 1479.56 ± 138.60 µm vs. 1281.71 ± 52.52 µm; paired *t*-test, p < 0.01), stronger corneal power (mean ± SD: 118.02 ± 2.54 D vs. 112.05 ± 2.96 D; paired *t*-test, p < 0.001), and reduced central corneal thickness (mean ± SD: 188.24 ± 6.08 µm vs. 196.78 ± 9.41 µm; Mann–Whitney *U*-test, p < 0.05). Correlation analysis of treated eyes showed that spherical-equivalent refractive error was significantly correlated with anterior segment parameters (Table 1): anterior chamber depth (Pearson’s r =  − 0.629, p < 0.01), corneal power (r =  − 0.707, p < 0.01), and central corneal thickness (r =  + 0.513, p < 0.05).

**Table 1:** Pearson’s correlation analysis between ocular refractive and axial components.

	SE	RA	CP	CA	CCT	ACD	VCD
SE	1	.669**	− .707**	.684**	.513*	− .629**	− .823**
RA		1	− .678**	.688**	0.146	− 0.237	− 0.282
CP			1	− .601*	− 0.394	0.482	0.386
CA				1	0.184	− 0.414	− .568*
CCT					1	− .624**	− .502*
ACD						1	.757**
VCD							1

Note: * p < 0.05. ** p < 0.01.

Abbreviations (Unit): SE, spherical equivalent (D); RA, refractive astigmatism (D); CP, corneal power (D); CA, corneal astigmatism (D); CCT, central corneal thickness (μm); ACD, anterior chamber depth (μm); VCD, vitreous chamber depth (μm).

**Figure 3:**
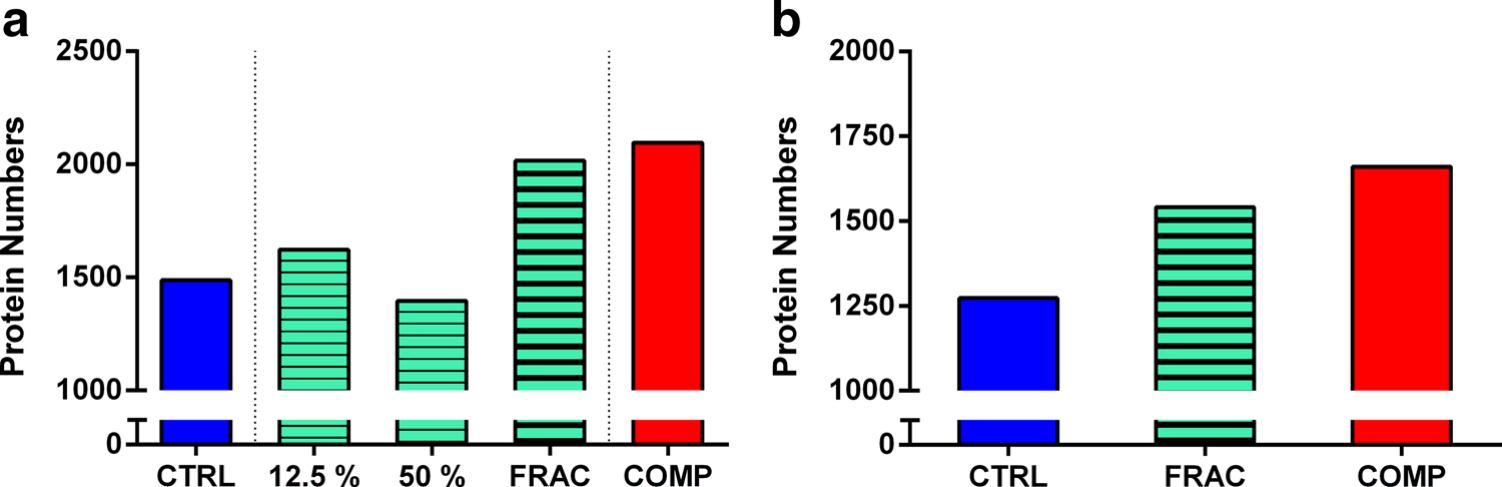
(**a**) Number of proteins derived from IDA-based ion spectral libraries with offline peptide fractionation. (**b**) Number of proteins derived by SWATH-MS analysis. Protein identification was performed at 1% FDR cutoff. CTRL = control library from the unfractionated control sample; 12.5% and 50% = fractionated libraries from offline peptide fractionated samples in two ACN gradients; FRAC = fractionated library by combining 12.5% and 50%; COMP = comprehensive library by combining FRAC and CTRL.

To generate a comprehensive proteome spectral library by IDA analysis, corneal tissue samples from both treated and fellow control eyes were pooled and fractionated or served as unfractionated controls. Fractionated samples, which combined two technical replicates, showed distinctive protein numbers and distributions at two gradient levels (1623 at 12.5% vs. 1396 at 50%; 817 shared; Fig. 3a and SFig 1A). Compared to the library of the unfractionated control sample, a noticeably larger number of proteins were detected through fractionated samples (2016 vs. 1487), with 764 proteins appearing exclusively after fractionation (Fig. 3a and SFig 1B). When combining fractionated and unfractionated control IDA libraries, a total of 2096 unique proteins (13,081 peptides) were discovered at 1% global false discovery rate (FDR), which can serve as a comprehensive library for the chicken cornea (COMP in Fig. 3a). Approximately 40% more proteins were found in this in-depth protein pool compared to the single unfractionated control library, suggesting that the coverage of further protein quantification is more effective by adopting the comprehensive library. The list of identified proteins was further classified using Panther GO into several sub-categories, which included “Molecular Function, Biological Function, Cellular Component, and Protein Class”. This classification was performed to visualize the overall composition of the comprehensive library, as well as to compare the proportion of proteins derived from peptide fractionation. As a result, 1347 out of 2096 proteins were mapped to Gene IDs for the analysis (see SFig 1 for an overview): (1) the major molecular functions were binding (GO:000,548; 36.9%), catalytic activity (GO:0,003,824; 37.4%), and structural molecule activity (GO:005,198; 9.0%); (2) biological functions were cellular process (GO:0,009,987; 34.8%), metabolic process (GO:0,008,152; 25.4%), and localization (GO:0,051,179; 13.5%); (3) regarding cellular components, proteins mostly performed their functions at cell (GO:0,005,623; 47.7%), organelle (GO:0,043,226; 27.2%), and protein-containing complex (GO:0,032,991; 11.9%); 4) three abundant protein classes were nucleic acid binding (PC00171; 14.2%), hydrolase (PC00121; 12.4%), and enzyme modulator (PC00095; 11.1%). To compare protein characteristics between the fractionated and unfractionated control samples, 1260 out of 2016 and 913 out of 1487 proteins, respectively, were mapped in Panther GO. As shown in Fig. 3, although the total protein numbers produced by these two methods were quite different, the proportion of proteins in the four GO categories (molecular function, biological process, cellular component, and protein class) were very similar (less than 3% differences).

**Figure 4:**
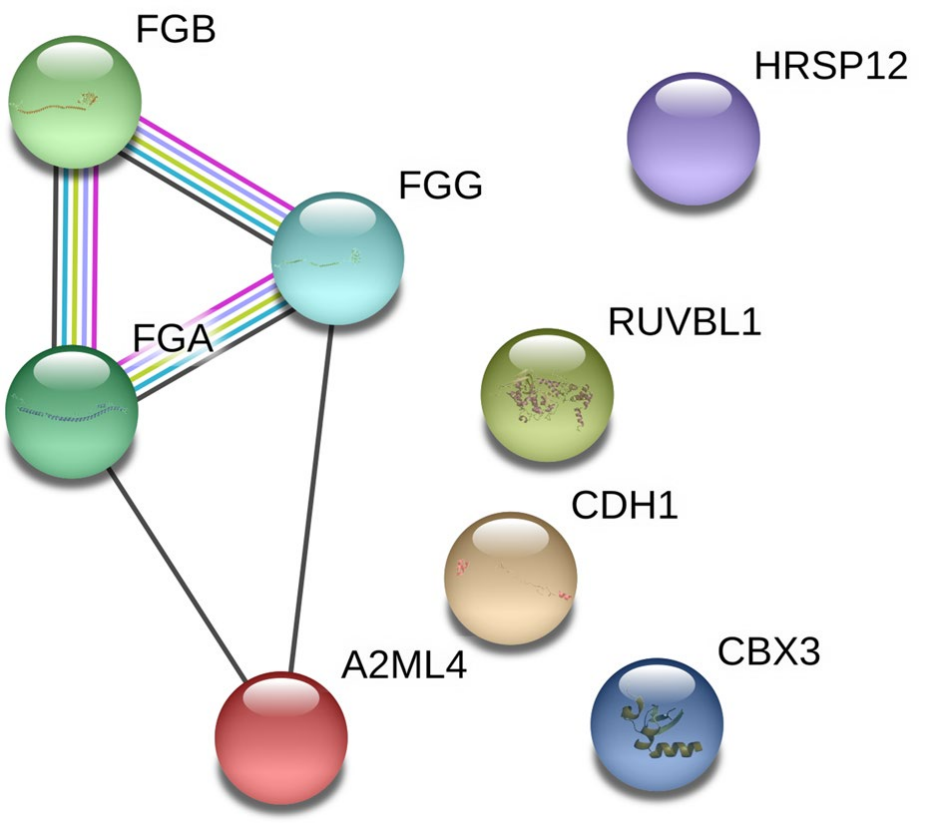
A diagram of protein–protein interaction derived from STRING. Fibrinogen chain alpha, beta, and gamma families (FGA, FGB, and FGG) have close interactions with each other and co-express with Alpha-2-macroglobulin-like 4 (A2ML4). Each colored line represents co-expression (black), interaction confirmed from the curated database (cyan), interaction confirmed from the experiment (pink), text mining (yellow), protein homology (light blue). Please refer to Table 2 for the abbreviation of annotated proteins.

Constructed ion-spectral libraries were used for SWATH-MS analysis to screen differentially expressed corneal proteins during high myopia development. The number of quantifiable proteins resulting from integrating different ion spectral libraries is shown in Fig. 3b, indicating that the coverage of libraries is crucial for increasing analysis depth in SWATH-MS analysis. Proteins IDs in comprehensive (n = 1660) and fractionated (n = 1541) libraries were extracted, and co-expressed proteins in both libraries were filtered. This step increases the reliability of results, although the number of quantifiable proteins is reduced (n = 1393). Subsequently, proteins with a minimum of two peptides were selected to minimize false-positive findings. Table 2 lists and highlights corneal proteins with statistically significant expression in treated eyes compared to fellow control eyes (> 1.2-fold differences with statistical significance of p < 0.05 in both comprehensive and fractionated libraries). After a week of FD treatment, it was found that three proteins were upregulated and five downregulated in treated corneas. These eight proteins were then analyzed using the STRING online pathway tool to investigate protein–protein interactions. As a result, Fibrinogen chain proteins (alpha, beta, and gamma) were observed to interact and co-express with Alpha-2-macroglobulin-like 4, while other proteins remained isolated (Fig. 4).

**Table 2:** Summary of differentially expressed proteins in corneal tissue after FD treatment.

No	Protein ID	Protein Description	Gene ID	Gene Description	Amino Acid Length	Mass (Da)	Fold changes (COMP)	P-values (COMP)	Fold changes (FRAC)	P-values (FRAC)
1	**A0A1L1RZS5**	**Reactive intermediate imine deaminase A homolog**	**RIDA**	**Reactive intermediate imine deaminase A homolog**	**139**	**14,821**	**1.3**	**0.021**	**1.3**	**0.013**
2	**E1C6M9**	**Cadherin-1**	**CDH1**	**Cadherin-1**	*887*	**97,755**	**1.2**	**0.016**	**1.2**	**0.041**
3	**F1N8Z4**	**RuvB-like helicase**	**RUVBL1**	**RuvB-like helicase**	**456**	**50,180**	**1.2**	**0.019**	**1.2**	**0.001**
4	E1C6J9	Thy-1 membrane glycoprotein	THY1	Thy-1 membrane glycoprotein	161	18,173	1.2	0.047	1.1	0.017
5	A0A1L1RIZ2	Calpain small subunit 2	CAPNS2	Calpain small subunit 2	248	27,411	1.1	0.031	1.2	0.032
6	A0A1I7Q419	40S ribosomal protein S12	RPS12	40S ribosomal protein S12	121	13,529	1.1	0.031	1.2	0.002
7	A0A1D5PKN8	Sulfurtransferase	MPST	Sulfurtransferase	297	33,223	1.1	0.002	1.1	0.026
8	F1NHH1	Uncharacterized protein	CSTB	Cystatin B	98	11,160	1.1	0.014	1.1	0.039
9	F1P360	Cytoskeleton associated protein 4	N/A	Cytoskeleton associated protein 4	499	56,823	1.1	0.043	1.0	0.043
10	R4GL78	Platelet-activating factor acetylhydrolase IB subunit beta	PAFAH1B2	Platelet-activating factor acetylhydrolase IB subunit beta	241	26,807	− 1.1	0.001	− 1.1	0.027
11	F1P2F0	Collagen alpha-3(VI) chain	COL6A3	Collagen alpha-3(VI) chain	3137	339,619	− 1.1	0.002	− 1.1	0.010
12	Q5ZKM2	Elongation factor 1-alpha	RCJMB04_10b5	Elongation factor 1-alpha	462	50,139	− 1.1	0.029	− 1.1	0.002
13	E1C7H6	Serpin family F member 1	SERPINF1	Serpin family F member 1	416	46,505	− 1.1	0.018	− 1.1	0.014
14	Q6QAZ9	Annexin	N/A	Annexin	342	38,500	− 1.1	0.046	− 1.1	0.037
15	**F1P4V1**	**Fibrinogen alpha chain**	**FGA**	**Fibrinogen alpha chain**	**783**	**87,295**	**− 1.2**	**0.002**	− **1.2**	**0.010**
16	**F1NK40**	**Uncharacterized protein**	**A2ML4**	**Alpha-2-macroglobulin-like 4**	**1474**	**163,339**	**− 1.2**	**0.001**	**− 1.2**	**0.0013**
17	**O93481**	**Chromobox protein (CHCB2)**	**CBX3**	**Chromobox protein (CHCB2)**	**174**	**19,777**	**− 1.2**	**0.030**	**− 1.2**	**0.045**
18	**F1NUL9**	**Fibrinogen beta chain**	**FGB**	**Fibrinogen beta chain**	**480**	**54,581**	**− 1.2**	**0.016**	**− 1.2**	**0.038**
19	**E1BV78**	**Uncharacterized protein**	**FGG**	**Fibrinogen gamma chain**	**438**	**49,955**	**− 1.3**	**0.008**	**− 1.3**	**0.005**

SWATH-MS was performed against two sets of in-depth libraries (COMP; comprehensive and FRAC; fractionated). Proteins with significant expression (> 1.2-fold differences) were highlighted.

## Discussion

This study yielded three novel findings: (1) chicken corneal proteome was reported for the first time; (2) the most comprehensive corneal proteome pools were successfully generated using IDA analysis with the offline peptide fractionation technique; (3) corneal proteins that may be involved in high myopia were screened using a novel SWATH-MS integrated with the extensive corneal proteome.

The proteome refers to the set of proteins containing biological information. By knowing and quantifying the proteome in areas of interest (cells, tissues, and organisms), the roles and functions of biomarker proteins in the disease process can be identified. However, although increasing efforts have been devoted to record and establish the complete proteome, there are only a limited number of human corneal proteome studies^23–25^ with corneal pathologies^44,45^. Although chicken has been used widely as an effective animal model for eye development and ocular pathologies, its corneal proteome had not been studied^35^. This study identified a total of 2096 highly confident proteins in chicken corneas using state-of-the-art proteomic approaches. This large proteome database provides an important foundation for future studies using chicken. A recent study has demonstrated the usefulness of merging published spectral libraries with other tissue specific libraries to discover more novel quantitative data from pre-acquired SWATH-MS files^18,46^, which would be technically impossible using conventional proteomics appraoch.

Even with the rapid advancement of proteomic analysis methods, understanding the entire proteome is challenging mainly because of the extremely complex protein structure and the differences in protein abundance. This leads to difficulties in detecting low abundant proteins as they can be masked by a few plentiful proteins^47^. Increasing the sensitivity of protein identification by lowering the detection threshold can be a solution. However, the trade-off of false-positive findings inevitably increases. A common alternative to address this limitation is using peptide fractionation strategies. These processes simplify the protein structure in an orthogonal direction and lower the number of proteins per MS analysis by assigning proteins into multiple fractions^48^, efficiently enabling the identification of a wide range of proteins. Currently, the majority of proteomic research applies online fractionation methods^49,50^, whereas offline methods are relatively rare, due to the extra manual steps and risk of protein loss during the process. Therefore, in this study, a high-pH reversed-phase peptide fractionation kit was adopted as an extra offline fractionation step^28^, which had the advantage of omitting the desalting step, in which a large amount of protein loss occurs. The detection of 2096 proteins indicates that this extended range of proteins was identified successfully using offline peptide fractionation prior to MS analysis. The current study only applied two acetonitrile levels (12.5% and 50% v/v ACN), so it is expected that a larger amount of proteins can be discovered if additional gradient steps are added as described in the manufacturer’s instructions.

Ocular tissue biomechanics play an important role in maintaining visual functions^51,52^. Instability in biomechanics is frequently associated with various shape-related ocular pathologies. Keratoconus, an abnormal protrusion of corneal shape, is related to reduced stiffness and altered biomechanics-related microstructures^53,54^. Interestingly, during myopia development, the sclera is known to experience tissue remodeling (weakening) and structural changes^55–57^ associated with ocular elongation, which is thought to due to remodeling of extracellular matrix (ECM) with molecular changes^57–63^. Both progressive keratoconus^64^ and myopia^65^ can be treated by tissue strengthening, supporting the role of ocular biomechanics in eye shape regulation. To date, several myopia-associated corneal biomechanical changes in humans have been reported^66–69^. However, the underlying mechanism of ocular biomechanics and their relationship with ocular morphologic changes and development are poorly understood. In our recent study^43^, reduced corneal biomechanics (softening) was accompanied by corneal steepening in experimentally induced highly myopic chicks. Since cornea and sclera are connected anatomically and share a similar collagen-dominated structural composition^70^, it is reasonable to assume that altered corneal biomechanics may be related to scleral biomechanics, particularly some biomarkers involved in ECM remodeling (e.g., matrix metalloproteinases-2; MMP-2^59,63^, tissue inhibitor of metalloproteinases-2; TIMP-2^63,71^, and transforming growth factor-beta 2; TGF-β2^63,72^).

This study applied a non-targeted discovery-based proteomic approach to screen differentially expressed corneal proteins in highly myopic eyes to understand whether ECM remodeling is also involved in corneal structural and biomechanical changes. As a result, eight corneal proteins were found to be expressed differentially (3 upregulated and 5 downregulated) in FD-treated highly myopic eyes. Of note, this list does not include genes previously reported to be involved in ECM remodeling (MMP-2, TIMP-2, and TGF-β2), the expression of these proteins either did not reach statistical significance (MMP-2 and TIMP-2) or was not listed (TGF-β2) in either the comprehensive or fractionated libraries. Nevertheless, it is interesting to note that there was a trend of MMP-2 up-regulation when interocular comparison was performed on an individual basis (fold changes: bird #1 =  + 1.36; bird 2 =  + 1.13; bird #3 =  + 1.30), indicating that averaging the values across animals may have masked this trend. Also, the increased expression of TIMP-2 (a fold change of + 1.36, p < 0.001) in this study is in agreement with a previous study showing up-regulation of TIMP-2 mRNA expression in FD-treated chick corneas^63^. Although DIA based SWATH-MS is known to be a stringent, consistent, and reproducible protein quantification tool, due to its novel peptide-centric scoring analysis^20^, there should be caution in interpretation of the results as the significantly differential expressions were observed only when a comprehensive library for SWATH-MS was applied, probably due to the low abundance of target proteins. Therefore, further targeted proteomic analysis with a capability of detecting changes in low abundant proteins, such as Multiple Reaction Monitoring High-Resolution (MRM-HR), will be necessary to confirm these findings^18,73,74^.

The key finding from the eight differentially expressed proteins is the down-regulation of fibrinogen family proteins (FGA, FGB, and FGG). Fibrinogen, a type of glycoprotein, has a series of functions, including blood clotting, fibrinolysis, wound healing, tissue repairing, and inflammatory response^75^. It also interacts with several cell types (fibroblast, platelet, and endothelial cell)^75^. During the coagulation process after tissue wounding, fibrinogen converts to insoluble fibrin. This fibrin then stabilizes platelets^76^ and activates the secretion of platelet-derived growth factors, stimulating fibroblasts to produce collagen, glycosaminoglycans, and proteoglycans^77^. However, this cascade of molecular events is related to an up-regulation of fibrinogen, in contrast to what was observed in this study (down-regulation) in highly myopic chick corneas. Fibrinogen deficiency could affect normal corneal wound healing^78^ but it is unclear how down-regulated fibrinogen is associated with myopia. It is possible that the up-regulated MMP-2 trend (see above) is related to down-regulated fibrinogen, based on the recent finding of fibrinogen as an inhibiting factor of MMP-2^79^. Interestingly, another down-regulated protein, alpha-2-macroglobulin-like-4, also inhibits a broad range of proteinases, including the MMP family^80,81^. It is worth noting that cadherin-1 (E-cadherin) was upregulated in the highly myopic chick corneas (Table 2). E-cadherin is part of a subfamily of classical cadherins, known for its involvement in cell–cell adhesions, cytoskeleton organization, and cell proliferation suppression^82^. Deficiency of E-cadherin could promote tumor progression^83^, as it inhibits invasion of tumor cells into ECM. In the cornea, E-cadherin is present in the epithelial layer^84^ and provides epithelial barrier function by increasing cell to cell interactions^85^. The weak association between corneal wound healing and E-cadherin expression^86^ suggests that the wound healing process is probably not involved in myopia-associated corneal remodeling. Evidence showing the close relationship between E-cadherin and the MMP family^87^ indicates the need to understand the role of E-cadherin in myopia progression. Nevertheless, all these findings support the involvement of MMP-2 in the corneal remodeling process in addition to its involvement in scleral remodeling reported in myopic animals^59,63^.

While this study provides fundamental resources of the chicken corneal proteome, several methodological limitations should be considered for future studies. Firstly, the FD paradigm was employed in this study as a first approach to understand potential molecular changes at protein levels in light of recent findings on altered corneal biomechanical properties in FD treated, highly myopic chicks^43^. This treatment paradigm induced high myopia and dramatic corneal structural changes within a short period of time, but also produced high inter-subject variability (Figs. 1, 2). This high inter-subject variability could potentially make some proteins with high inter-subject variation undetectable (see MMP-2 discussion above). Mainly because the lens induction paradigm produced much less inter-subject variation compared to FD, applying proteomic analyses on lens-induced myopia (LIM) chicks may confirm or even extend the list of differentially expressed proteins. To date, several proteomic studies using LIM treatment of chicks have been reported (retina^26,88^ and vitreous^27^), supporting the efficacy of this treatment paradigm. Secondly, quantifying protein expression at a single time point has obvious limitations. The time point chosen (one week of FD commencing on P5) was based on the significant changes in corneal structural and biomechanical parameters reported recently^43^. However, caution should be applied when attempting to relate these differential expressions to the cause or effect of the myopia development based on a single time point. A significant knowledge gap remains on the spatial–temporal changes in the molecular pathways regulating myopia development. Thirdly, inadequate sample size may affect the quality of results by restricting the number of technical and biological replicates, which is essential for reliable quantitative analysis. It was observed in the current study that protein concentrations in a single cornea are considerably low compared to other ocular tissues (e.g., retina). Sample pooling could be a possible solution to secure enough samples. However, this could limit the statistical power of biomarker detection by altering mean and standard deviation of analytes^89,90^.

In conclusion, our study documented, for the first time, a large corneal proteome of chicken by applying novel bioinformatics analysis with offline peptide fractionation. Differentially expressed corneal proteins in highly myopic eyes using a SWATH-MS strategy suggest that molecular changes at protein level are involved in corneal remodeling at least at this time point. These results provide fundamental information for future corneal research, especially those using chicken as an animal model for myopia development.

## Materials and methods

### Animals

Eight White Leghorn chicks (Gallus gallus domesticus) were raised in the Centralized Animal Facility of The Hong Kong Polytechnic University. During the husbandry, chicks had ad libitum access to food and water. The photoperiod of the animal room was automatically controlled at a 12:12 h light–dark cycle. All experiments were conducted in accordance with the ARVO Statement for the Use of Animals in Ophthalmic and Vision Research and ARRIVE guidelines (Animal Research: Reporting of In Vivo Experiments). The protocol for this study was approved by Animal Subjects Ethics Sub-Committee (ASESC 16–17/22) of The Hong Kong Polytechnic University.

### Treatments & biometric measurements

Chicks were reared from day 5 post-hatching (P5) with their right eyes covered with plastic-molded translucent diffusers for 7 days to induce monocular form deprivation myopia (FD) while left eyes remained untreated to serve as contralateral controls. It has been suggested that FD by diffuser treatment imposes significantly reduced retinal image contrast, so eyes may be deprived of normal visual feedback to control its growth^91^. This treatment typically results in axial myopia that is mainly attributable to elongated vitreous chamber depth, accompanied by anatomical and biological changes in choroid, retina, and sclera in chicks^43,92^, resembling ocular changes in human axial myopia^93^. Similarities in ocular responses to this classic experimental protocol across various animal species^94–96^, including humans^97,98^, suggest that FD is one of the most effective models for myopia research^99^. At the end of the treatment period (P12), biometric measurements, including corneal videokeratography, ocular axial dimensions, and refractive status were performed as described in a previous study^43^. Briefly, a custom made videokeratography system (VKS) was used to measure corneal astigmatism and corneal power^100^. Approximately 600 consecutive corneal images were captured by a CCD camera with a frame rate of 60 frames per second (Guppy GF 046B, Allied Vision, Germany) after aligning the pupillary center with Placido rings. The distance between adjacent reflected concentric rings was used to exclude images of accommodated cornea (constricted Placido rings), and around four to five images per eye were manually chosen for image analysis using a custom-written MATLAB algorithm. The corneal radii of curvature and astigmatic components (J0 and J45, calculated by Power vector analysis) derived from these images were averaged^101^. Chicks were then anesthetized with isoflurane inhalation (1.5% to 2.0% with oxygen) to collect ocular axial dimensions measured by a high-frequency A-scan ultrasonographer (GE Panametrics, U.S.). Three measurements per eye, each measurement consisting of 30 data sets were conducted and averaged after manually identifying peaks representing the inner ocular surfaces^102^. Then, a minimum of three refractive error measurements was carried out per eye using a modified Hartinger refractometer^40^ and averaged for spherical equivalent, J0, and J45 astigmatic components.

### Tissue collection

**Figure 5:**
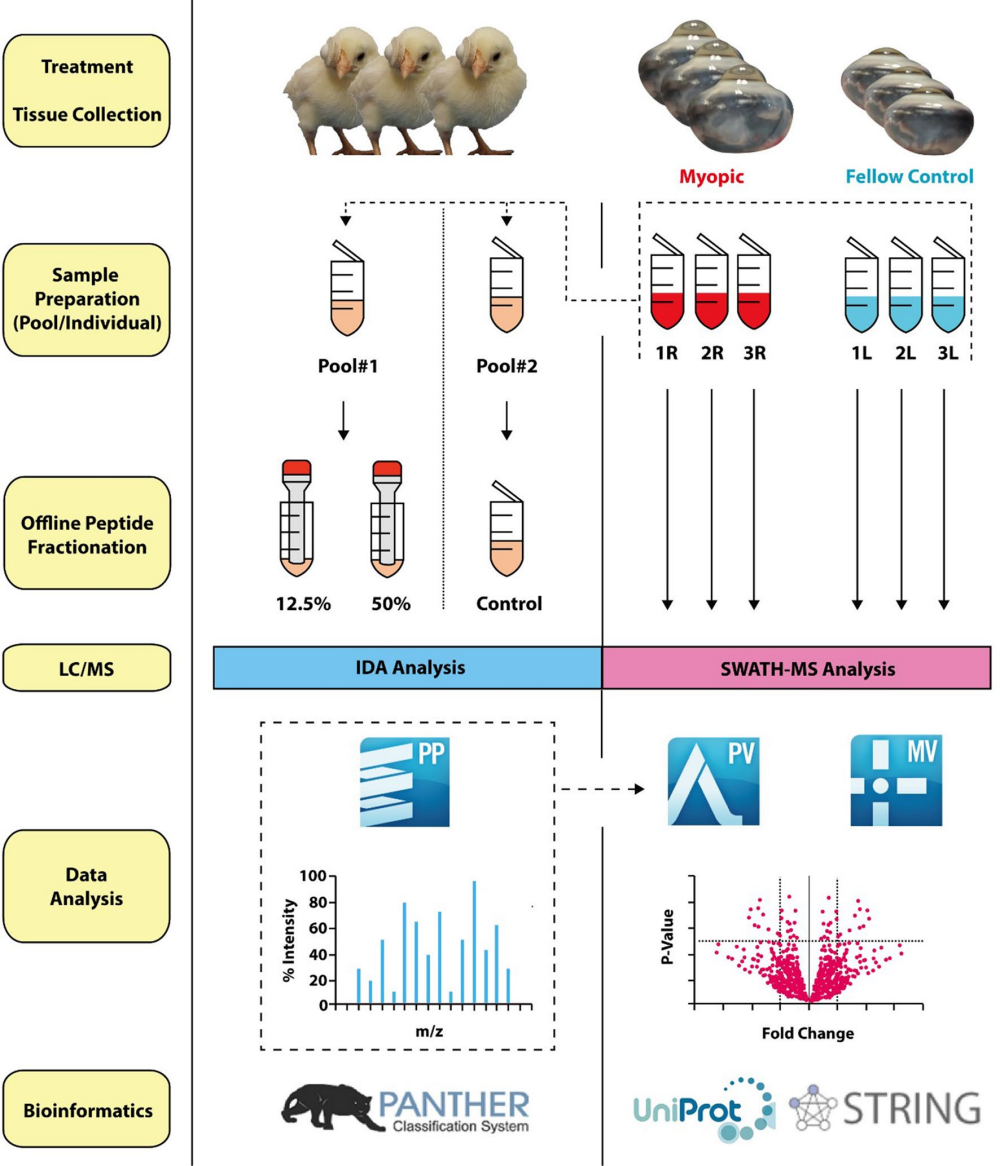
A schematic diagram showing the workflow of proteomic analysis. The experiments were divided into two phases: Left) generating ion spectral library using IDA analysis integrated with ProteinPilot (PP), and Right) discovery of differentially expressed proteins via SWATH-MS analysis integrated with PeakView (PV) and MarkerView (MV).

Detailed procedures of proteomic analysis workflow have been described in the proteomics data journal^29^ and are briefly summarized in Fig. 5. High inter-subject variability in refractive development and ocular biometries are common in FD treatment, which may affect the outcome of proteomic analysis. Therefore, only the three of the eight chicks that developed high myopia (> 20 D), with similar interocular changes in the corneal radius of curvature (< –7%) and axial length (> + 9%), were used. After chicks were euthanized by carbon carbon dioxide asphyxiation^43^, corneal tissue samples of 4-mm diameter were collected and stored in liquid nitrogen.

### Sample preparation

Corneal tissues were homogenized with 100 µL of a customized lysis buffer [30 mM tris-HCI (pH 8.5), 7 M urea, 2 M thiourea, 2% (v/v) CHAPS, 1% (v/v) ASB14 with a protease inhibitor cocktail (Complete, Roche Molecular Systems, U.S.)], followed by protein concentration measurement using a 2-D Quant Kit (GE Healthcare, U.S.). To build proteome libraries (IDA analysis), an identical amount of proteins from each sample was extracted, equally pooled (total = 13 µg), and fractionated by using a kit (Pierce High-pH Reversed-phase Peptide Fractionation Kit, Thermo Fisher Scientific, U.S.) with two-step gradient elution solutions of either 12.5% or 50% (v/v) ACN dissolved in 0.1% (v/v) trifluoroacetic acid (TFA). For protein quantification (SWATH-MS analysis), each sample was digested with trypsin (1 µg per 25 µg protein amount), and contaminant extraction was performed using a cleanup kit (Oasis HLB Sorbent Cartridge, Waters, U.S.) to enhance the sample quality of the extracted peptides^103,104^. Then the samples were re-suspended by adding 0.1% (v/v) formic acid.

### Protein identification by IDA

A hybrid TripleTOF 6600 quadrupole Time-of-Flight mass analyzer (Sciex Framingham, MA) connected to a nano LC415 was applied for proteomic data acquisition. Three types of pooled samples (2 µg each) with two technical replicates were used to generate reference proteome libraries: high-pH reversed-phase fractionated (12.5% and 50% v/v ACN; see details in the previous study^29^) and unfractionated control. Acquired raw database (.wiff) were imported and integrated in ProteinPilot software (Version 5.0.1, Sciex Framingham, MA) with Paragon Algorithm search engine^105^ (i.e. comprehensive = fractionated + unfractionated control; fractionated = 12.5% + 50% v/v ACN; and unfractionated control). Only proteins with high confidence (1% global FDR) were considered for further bioinformatics analysis.

### SWATH-MS

Six samples (3 myopic and 3 contralateral fellows; 2 μg each) with two technical replicates were used for quantification. PeakView software (Version 2.1, Sciex Framingham, MA) with two reference proteome libraries, produced from IDA analysis (comprehensive and fractionated; see details in Protein Identification by IDA) were used to match the corresponding peptide fragment peaks from raw data (.wiff). Peptide confidence and FDR thresholds were given at 95% and 1% respectively. Resulting data were exported to MarkerView software (Version 1.3.1, Sciex Framingham, MA) for normalization (most likely ratio method; MLR^106^), followed by statistical analysis (unpaired *t*-test). Raw data generated from IDA analysis and SWATH-MS are available at Peptide Atlas public repository (http://www.peptideatlas.org/PASS/PASS01410) for public access.

### Bioinformatics analysis

Identified proteins were categorized by gene ontologies (cellular component, molecular function, and biological process) through the PANTHER (protein analysis through evolutionary relationships) online classification system (http://www.pantherdb.org/)107. Significantly differentially expressed proteins found in SWATH-MS analysis were visualized using a Venn diagram, and the list of proteins shared in multiple libraries was further investigated using Uniprot protein database to determine their functional information (http://www.uniprot.org/)108. Additionally, a protein–protein interaction network was analyzed using STRING V11 (online pathway analysis tool; https://string-db.org/)109.

### Statistical analyses

All statistical analyses were performed using IBM SPSS (version 21.0.0, IBM, U.S.) and Microsoft Excel (version 2016, Microsoft Corp, U.S.). After confirmation of data normality by Shapiro–Wilk test, refraction and ocular biometric differences between treated and untreated contralateral eyes were compared by paired t-test. Pearson’s correlation analysis was further conducted to explore the relationship between ocular refractive and axial components. The significance level for all tests was set at 5%.

## Supplementary Information

Supplementary Information 1.
