# Supplementary material for: Corneal proteome and differentially expressed corneal proteins in highly myopic chicks using a label-free SWATH-MS quantification approach
Source: Sci Rep. 2021 Mar 9;11:5495. doi: 10.1038/s41598-021-84904-4 (PMC7943770; doi:10.1038/s41598-021-84904-4)
Supplement: Supplementary file 1 — Supplementary Information 1. [file 41598_2021_84904_MOESM1_ESM.docx]

**Corneal proteome and differentially expressed corneal proteins in highly myopic chicks using a label-free SWATH-MS quantification approach**

Byung Soo Kang^1*^, Thomas Chuen Lam^1^, Jimmy Ka-wai Cheung^1^, King Kit Li^1^, Chea-su Kee^1*^

^1^ School of Optometry, The Hong Kong Polytechnic University, Hong Kong SAR, China

*Corresponding authors; Equal contribution

**Mr Byung Soo Kang**

School of Optometry, The Hong Kong Polytechnic University, Hong Kong

Tel: (852) 9625 4515 E-mail: [bs.kang@polyu.edu.hk](mailto:bs.kang@polyu.edu.hk)

**Dr Chea-su Kee**

School of Optometry, The Hong Kong Polytechnic University, Hong Kong

Tel: (852) 2766 7941 E-mail: [c.kee@polyu.edu.hk](mailto:c.kee@polyu.edu.hk)


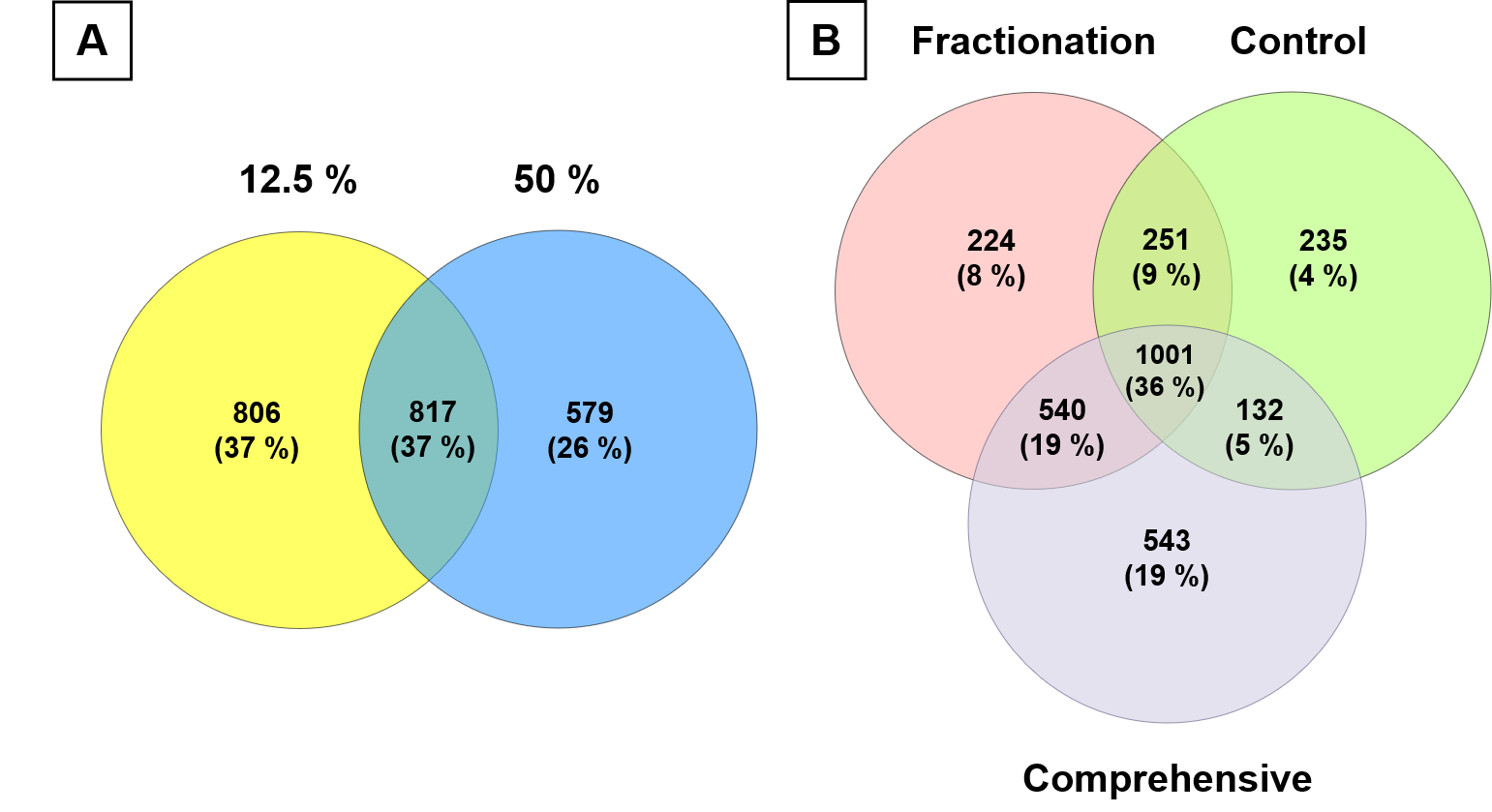


**Supplementary Figure 1**. Venn-diagrams showing: A) the number of detected proteins from two gradient eluting solutions (12.5 % and 50 % ACN) using derived fractionated libraries; B) protein distributions across all libraries. The percentage in parentheses refers to: (the number of proteins/total number of proteins) * 100.





**Supplementary Figure 2**. The percentage of corneal proteins categorized by molecular functions (A), biological processes (B), cellular components (C), and protein classes (D). Black, red, and blue bars represent comprehensive, fractionated and unfractionated control libraries, respectively.
